# Perceived barriers and enablers for preventing the spread of carbapenem producing gram-negative bacteria during patient transfers: a mixed methods study among healthcare providers

**DOI:** 10.1186/s12879-019-4684-x

**Published:** 2019-12-11

**Authors:** Eline van Dulm, Wendy van der Veldt, Katja Jansen-van der Meiden, Gerry van Renselaar, Lian Bovée, Jeanette Ros, Udi Davidovich, Yvonne van Duijnhoven

**Affiliations:** 1 Department of Infectious Diseases, Public Health Service Amsterdam, Nieuwe Achtergracht 100, 1018 WT Amsterdam, the Netherlands; 2 Department of Infectious Diseases, Public Health Service Kennemerland, Haarlem, the Netherlands; 3 Department of Infectious Diseases, Public Health Service Flevoland, Lelystad, the Netherlands

**Keywords:** Carbapenem producing gram-negative bacteria, Carbapenem producing *Enterobacteriaceae*, Communication, Knowledge, Healthcare providers

## Abstract

**Background:**

Antimicrobial resistance (AMR) increasingly threatens public health. Carbapenem-producing gram-negative bacteria (CPB) pose the biggest threat. The risk for CPB spread is heightened during the transfer of a CPB-positive patient between different healthcare institutions or healthcare providers. We aimed to gain insight into the frequency of CPB-positive patients in the Dutch provinces of Noord-Holland (NH) and Flevoland (FL). Secondly, we aimed to obtain a deeper understanding of the communication between healthcare providers during transfers of CPB-positive patients and explore possible communication-related risk situations for CPB spread.

**Methods:**

This mixed-methods study consisted of a quantitative and qualitative section. For the quantitative section, 14 laboratories that provide diagnostics in NH and FL voluntarily reported carbapenem-producing *Enterobacteriaceae* (CPE) positive patients between February 2018 and February 2019. Additionally, two laboratories reported carbapenem-resistant *Acinetobacter spp.* (CRA) and carbapenem-resistant *Pseudomonas aeruginosa* (CRP) positive patients. For the qualitative section, healthcare providers of reported patients were interviewed about information exchange during patient transfers, precautionary measures and knowledge and beliefs concerning CPB.

**Results:**

In total, 50 CPE-positive, 10 CRA-positive and 4 CRP-positive patients were reported during the inclusion period. Eighteen index-specific and 2 general interviews were conducted with 20 different care providers of 9 patients. The interviews revealed that, in most cases, information concerning the patient was transferred timely, but often a standardized method for sharing the information within and between institutions was lacking. Factors that enhanced care providers’ motivation to adhere to precautionary measures were taking responsibility for the health of other patients, (pregnant) colleagues and for ones own health. Factors that reduced motivation were not acknowledging the relevance of the precautionary measures, a perceived negative impact of the measures on patients’ recovery, differences in precautionary measures between healthcare settings and incomprehension for changes in precautionary measures.

**Conclusions:**

CPB-positivity occurred more frequently than expected in the Dutch provinces of NH and FL. Standardizing the transference of information concerning CPB-positive patients, implementing transmural agreements, training personnel on CPB knowledge and procedures, launching a national website on CPB and assigning one or several designated employees for CPB within healthcare institutions could improve communication between healthcare providers and thereby decrease the risk of CPB transmission.

## Background

Antimicrobial resistance (AMR) is a growing worldwide problem and an increasing public health threat [1]. The World Health Organization (WHO) warns that new resistance mechanisms are emerging and that our ability to treat common infections is being threatened [2]. It is estimated that in 2015, approximately 33,110 deaths attributable to infections with antibiotic-resistant bacteria occurred in the European Union [3].

The Netherlands belongs to the countries in Europe with the least infections caused by antibiotic resistant bacteria [3, 4]. It is estimated that in 2015, approximately 5000 infections with antibiotic-resistant bacteria occurred in the Netherlands, which resulted in 206 deaths attributable to AMR [3]. Although there is still room for improvement, the current AMR situation in the Netherlands is encouraging. Firstly, physicians in the Netherlands are generally reserved in prescribing antibiotics [5]. Secondly, the use of antibiotics in livestock has decreased with approximately 64% between 2009 and 2016 [6]. In addition, Dutch hospitals actively combat AMR through the Dutch search and destroy policy for methicillin-resistant *Staphylococcus aureus* (MRSA) [7] and the so called ‘A-teams’ (antimicrobial stewardship teams) for improving antibiotic stewardship of specialist physicians in hospital settings. Finally, there are multiple professional guidelines for preventing spread of AMR for both inpatient and outpatient settings [8]. Nonetheless, the infection pressure of resistant bacteria from other countries [9], the environment, the food chain and within healthcare settings remains, occasionally resulting in outbreaks of resistant bacteria [10–12]. From April 2012 to May 2018, a total of 212 outbreaks of resistant bacteria that were a threat to the continuity of care have been reported to the national early warning and response meeting of hospital-acquired infections and antimicrobial resistance (SO-ZI/AMR) in the Netherlands. Of these outbreaks, 44 were reported in the provinces of NH and FL [13]. These outbreaks highlight the need for insight into risk situations for the spread of AMR to prevent increasing morbidity and mortality due to (further spread of) AMR.

Carbapenem-producing *Enterobacteriaceae* (CPE), carbapenem-resistant *Acinetobacter spp*. (CRA) and carbapenem-resistant *Pseudomonas aeruginosa* (CRP) belong to the group of multidrug-resistant (MDR) bacteria and are categorized as priority-1 (CRITICAL) bacteria by the WHO [14]. In healthcare, these carbapenem-producing gram-negative bacteria (CPB) (as their susceptible analogous) most commonly are directly transmitted from patient to other patients through contaminated hands or through contaminated hands of healthcare workers due to physical contact with the patient. In addition, transmission can occur indirectly, through shared equipment or contaminated environmental surfaces [15]. CPB are prevalent worldwide, however differences in prevalence exist between and within continents and countries [16–20]. In the Netherlands, patients are occasionally diagnosed with CBP, mainly following hospital admission abroad [21]. However, based on data submitted by 28 Dutch laboratories to the Infectious diseases Surveillance System-Antibiotic Resistance (ISIS-AR), the overall prevalence of gradient test confirmed CPE has slightly increased (from 0.02% in 2014 to 0.05% in 2018 for *Escherichia coli* and from 0.25 to 0.52% in *Klebsiella pneumoniae*) [22]. Even though CPE are still relatively rare in the Netherlands, they pose the biggest threat to public health, since infections with these bacteria leave very few therapeutic options [23, 24] and are often associated with prolonged hospitalization and increased mortality [25]. Therefore, in April 2018, the Dutch minister of Health, Welfare and Sports decided that CPE should become mandatory notifiable as a category C item (to be reported on within one working day following diagnosis by the head of the laboratory to the public health service (PHS) in the Netherlands) [26, 27]. This has taken effect as of July 1st 2019.

Various (medical) risk factors exist for CPB acquisition among hospitalized patients, the most important ones being the use of medical devices and carbapenem use [28]. However, a situation in which the risk for the spread of CPB is also heightened, is during the transfer of a CPB-positive patient between different healthcare institutions or healthcare providers. When information on CPB-positive patients is inadequate or not shared timely, precautionary measures to prevent the spread of these bacteria are hampered. For healthcare providers, both in the inpatient ‘cure’ settings as in the outpatient ‘care’ settings, actual knowledge of precautionary measures and existing guidelines for CPB containment is increasingly important. Especially in the context of elderly living longer at home independently and being discharged sooner from hospitals nowadays. Due to these developments, the number of CPB-positive patients requiring care in outpatient settings is increasing. It is assumed that a knowledge gap concerning CPB exists in outpatient settings and that therefore an increase in knowledge and awareness concerning CPB is required of healthcare providers in these settings.

Therefore, we firstly aimed to gain insight into the frequency of CPB-positive patients in the Dutch provinces of Noord-Holland (NH) and Flevoland (FL) between February 2018 and February 2019 by means of a quantitative study component. Secondly, by means of a qualitative study component, we aimed to obtain a deeper understanding of the communication process between healthcare providers during transfers of CPB-positive patients and explore possible communication-related risk situations for the spread of CPB. The results of this study provide insight in the magnitude of diagnosed CPB-positive patients and the communication between healthcare providers involved. Therefore, the study could contribute to the formulation of transmural agreements between institutions and healthcare providers regarding AMR and patient transfers in the Netherlands.

## Methods

### Recruitment and sample

The Dutch provinces of NH and FL are divided into 6 PHS regions, in which a total of 14 laboratories provide diagnostics. All were approached to participate and agreed to voluntarily report CPE-positive patients anonymously to their regional PHS. Contact persons of the 6 PHS subsequently reported the cases to the research team of the study. Additionally, two laboratories voluntarily reported CRA/CRP-positive patients. During the recruitment period from February 2018 to February 2019, a total of 64 patients were reported to the research team. For each reported patient, one of the research nurses contacted the reporting medical microbiologist of the laboratory to obtain the name of the responsible healthcare provider of the patient (the one requesting the diagnostics from the laboratory and being the formal responsible for treatment and care). The nurse then contacted this responsible healthcare provider and requested them to obtain the patient’s informed consent for following the patient’s route in the healthcare system. After written informed consent was obtained from the patient, the research nurse planned an interview with the previously contacted primary responsible healthcare provider of the patient. Additionally, based on information obtained from the initial healthcare provider, other healthcare providers involved in the care of the CBP-positive patient were contacted by the research nurse and asked to participate (up to 5 healthcare providers per patient). Nurses asked when the patient was expected to leave the healthcare setting (e.g. transferred to another healthcare organization, or discharged), to be able to contact potential successive healthcare providers. To limit healthcare providers’ time investment, verbal oral consent for participation was obtained at the start of the telephone interview.

### Inclusion criteria

CBP-positive patients that were 18 years or older and received care (either in an institution or at home) were eligible for participation in the qualitative data collection. Healthcare providers were excluded when they were not able to remember the patient or when the patient was reported to the regional PHS ≥2 months after the CBP diagnosis.

### Quantitative section

#### Laboratory detection

All participating laboratories perform CBP diagnostics according to the guideline of the Dutch Society for Medical Microbiology (in Dutch: Nederlandse Vereniging voor de Medische Microbiologie (NVMM) [29] and are ISO 15189 accredited [30]. This prescribes the detection of carbapenemase production as a two-step procedure, of which the first step (screening) is performed by the diagnosing laboratories and the second step (phenotypic and genotypic confirmation) is mostly performed by the Dutch national institute for public health and the environment (RIVM). In short, screening occurs with a selective plate for CBP. When suspicious isolates prove to have a mean inhibitory concentration (MIC) > 0,25 mg/L for meropenem, the elevated carbapenem MIC is confirmed with antibiotic gradient on a strip method. In most participating laboratories, when the strip method confirms a MIC > 0.25 mg/L, a PCR on known carbapenemase genes is performed and the strain is sent to the RIVM for phenotypic and genotypic confirmation. Some laboratories perform their own phenotypic confirmation by means of a CIM (carbapenem inactivation method) test [31].

#### Quantitative analyses

CBP cases were reported to the local PHS by email. Notifications included the name, sex and date of birth of the patients as well as the detected micro-organism. The local PHS forwarded the notification to the research team and KJ and GR processed the information in an excel sheet with all notifications. An index number was assigned to each patient and the qualitative data were obtained and processed by making use of the same index number. Descriptive analyses were performed to obtain the patients’ age at time of reporting and the distribution of the different types of CBP that were detected. All analyses were conducted with Stata 13 (StataCorp., College Station, Texas, USA).

### Qualitative section

#### Interview procedures

Semi-structured interviews were chosen as method of investigation, as they allow flexibility to explore new themes and can generate richer thematic data [32]. All interviews were conducted in Dutch and were performed and conducted by telephone to minimize time investment of the healthcare providers. Two research nurses (KJ and GR) with previous qualitative interview experience conducted the interviews. At the beginning of the interview, the study was explained, patient and healthcare provider data were verified and oral informed consent of the healthcare providers was obtained. The following subjects were addressed during the interviews: information exchange during patient transfers (what information was shared, in what manner, when, by who); precautionary measures (what measures were taken, on what grounds, were there barriers in applying these measures); the availability of guidelines; the healthcare provider’s knowledge concerning CBP and need for more information concerning CBP; other healthcare providers that were involved in patient care (to plan additional interviews). An additional file shows the interview guide that was used (Additional file 1). The main goal of the interviews was to gain insight in the handlings of CBP in NH and FL and to reveal the information exchange between healthcare providers of CBP-positive patients, including barriers and enablers of patient related communication.

#### Qualitative analyses

To limit researchers’ time investments, all interview recordings were transcribed by a processing agency. Consequently, the researchers entered the transcripts into a database using qualitative data analysis software (MAXQDA 2018). A descriptive content analysis was performed by two researchers (WV and ED) in an inductive manner. Subsequently, the two researchers discussed the content of the interviews and reached consensus on interpretation and important findings.

#### Ethical framework

This study was approved by the Medical Ethical Committee of the Academic Medical Centre of Amsterdam, the Netherlands (W17_384). In order to maximize confidentiality, all possible personal identifiers were removed from interview transcripts. Interview transcripts were only accessible to researchers from the research team. All respondents were able to withdraw consent to participate in the study at any time without clarification.

## Results

### Quantitative section

#### Reported CBP-positive patients

In total, 50 CPE-positive patients were reported between February 2018 and February 2019. Additionally, two laboratories reported 10 CRA-positive patients and 4 CRP-positive patients. This resulted in a total of 64 patients, approximately two cases per 100,000 inhabitants of NH and LF. Fifty-nine patients (92%) were reported by seven laboratories and five patients were reported by another Dutch research group working on a CPE study. Of the reported patients, the majority was male (58%) and the median age at time of reporting was 69 (IQR 54–76). During the inclusion period of this study, an outbreak of the CPE *Citrobacter freundii* occurred, which resulted in 22 *Citrobacter freundii* positive patients from 1 laboratory being included in our study. Therefore, most patients included in the study were diagnosed with *Citrobacter freundii* (36%), followed by *Escherichia coli* (17%) and *Klebsiella pneumoniae* (16%)*.* Excluding the 22 outbreak patients, patients were most often diagnosed with *E. coli* (11/42, 26%), *K. pneumoniae* (10/42, 24%) and *Acinetobacter spp.* (10/42, 24%).

**Fig. 1:**
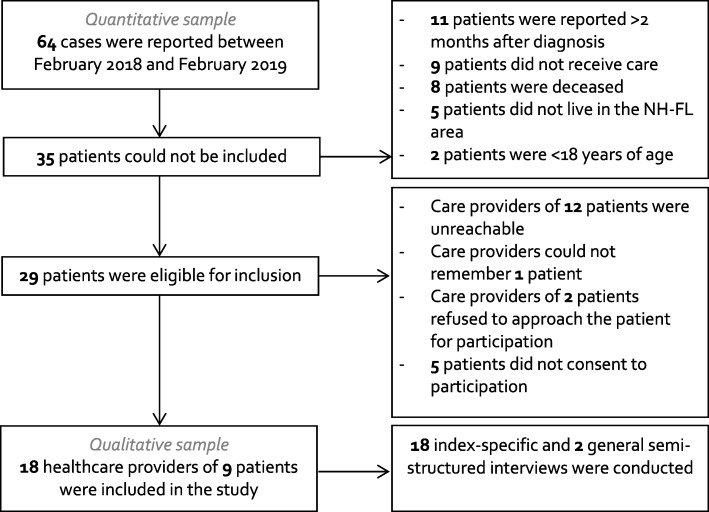
Flowchart of inclusion and exclusion of CPE/CRA/CRP-positive patients and their healthcare providers. Abbreviations: NH – Noord-Holland; FL – Flevoland

Of the 64 cases, 35 patients (55%) were not eligible for inclusion: 11 patients (17%) were reported at the PHS ≥2 months after diagnosis, nine patients (14%) did not receive care during their diagnosis, eight patients (13%) were deceased, five patients (8%) lived outside of the NH-FL area and two patients (3%) were < 18 years of age. Of the 29 eligible patients (62% male, median age at time of reporting 67, IQR 54–75), in 12 patients (41%), care providers could not be reached within the study timeframe despite at least five attempts at different days and time periods. Furthermore, one patient (3%) was excluded because the care provider could not remember the patient, care providers of another 2 (7%) patients refused to approach the patient for participation and five patients (17%) did not consent to participation. Finally, eight CPE-positive patients and one CRA-positive patient consented to approach their healthcare providers. Of these nine patients included in the qualitative study, six were male (67%) and median age at time of reporting was 75 (IQR 59–78 years). Figure 1 shows the flowchart of the inclusion and exclusion of the patients.

### Qualitative section

#### Sample characteristics

In total, 18 index-specific and 2 general interviews (mean duration 20 min, ranging from 10 to 35 min) were conducted with 20 different care providers (70% female). For the index-specific interviews, the time from the patient being reported to the first interview ranged from 37 to 100 days. Eight of the 20 interviewees (40%) were specialist doctors (6 were hospital-based, 2 were general practitioners and 1 was based in a revalidation center). Eight interviewees (40%) were registered nurses (1 was hospital-based, 3 were employed at a revalidation center, 3 were nurses in home-based care and 1 was employed at a nursing home) (Table 1).

**Table 1:** Qualitative sample characteristics

Respondent number	Type of institution	Profession	Sex	Index number
1.	Revalidation center	Nurse	Female	1
2.	Home based care	Nurse	Female	2
3.	Revalidation center	Nurse	Female	3
4.	Revalidation center	Physical therapist	Female	
5.	Revalidation center	Occupational therapist	Male	
6.	Hospital	Physician	Male	4
7.	Revalidation center	Physician	Male	
8.	Home based care	Nurse	Female	
9.	Hospital	Medical fellow	Female	5
10.	Nursing home	Nurse	Female	
11.	Hospital	Nurse	Female	
12.	Hospital	Physician	Male	6
13.	Hospital	Physician	Female	
14.	Revalidation center	Healthcare assistant	Male	7
15.	Home based care	Nurse	Female	
16.	General practice	General practitioner	Female	
17.	Revalidation center	Nurse	Female	8
18.	General practice	General practitioner	Female	9
19.	Hospital	Infection prevention specialist	Female	None
20.	Hospital	Surgeon	Male	None

#### Complex care networks

**Fig. 2:**
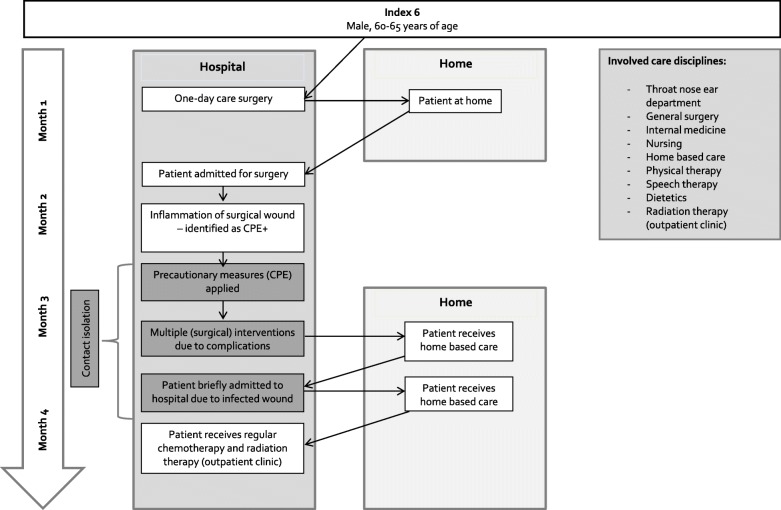
Example of cascade of care and involved care disciplines. Abbreviations: CPE – Carbapenem-producing *Enterobacteriaceae*

The personal cascades of care varied considerably between patients. For all 9 included patients, multiple care providers within one institution were involved in the care of the patient. In 70% of the included cases, multiple care providers within one institution and from different institutions were involved. Furthermore, patients were often transferred from one institution to another or discharged from an institution within a short period of time. Figure 2 provides an example of the complex care network and the abundance of involved care disciplines of one of the included patients, demonstrating the theoretical potential of spread of CPB within and between healthcare settings and care providers.

#### Interview findings

##### Communication

The interviews revealed that, in most cases, information concerning the CPE/CRA-positive patient was shared timely (e.g. before the patient was transferred or immediately after the patient was found to be CPE/CRA-positive). Three care providers indicated that information was not accessible in time which resulted in a state of confusion.

*“Yes, I can remember that it [the sharing of information] was at a later time point, that I had already been there [the patient] once [after the patient was diagnosed with CPE] and that I did not apply contact isolation that first time.” (respondent number 1, general practitioner).*

Methods for the transference of information were several and differed between and within institutions. Communication methods entailed transfer letters, telephone, e-mail, face-to-face conversations, work meetings and electronic transfer dossier (POINT). For some patients, multiple methods of information transference were used (information was shared face-to-face and through email, information letter and personal documents of patient). In almost all cases, the CPE/CRA-positivity of patients was made known by signs on the door of the patients’ room and by a pop-up in the electronic patient dossier (EPD). As mentioned before, most of the healthcare providers received the information in time and were satisfied with the information. This was especially true for healthcare providers in institutional care, since these institutions often had a designated employee responsible for infection prevention. In these cases, the infection prevention measures in case of a CPE/CRA-positive patient were known and easily applied according to the interviewees.

The interviews also uncovered several suboptimal aspects of the communication about CPE/CRA-positive patients. In almost all interviews, the transference of information proved not to be clearly arranged, since no standardized method and route for sharing the information within and between institutions exist. This resulted in confusion among care providers and most of them were not able to reproduce how and when the information concerning the CPE/CRA-positive patient was transferred to them. Furthermore, sharing information concerning CPE/CRA-positivity and aligning infection prevention measures did not routinely include disciplines or departments such as cleaning and food distribution. For many care providers, this was the first time they encountered a CPE/CRA-positive patient in their career. Therefore, in many cases, care providers felt the need to look up extra information on CPE/CRA and infection prevention measures. In some interviews, a passive attitude towards these infection prevention measures was observed: care providers did only acknowledge that precautions had to be taken when seeing a sign on the door of a patient or when gloves were placed in the room of a patient.

*“The patient told me that the home-based care nurses wore aprons with gloves, and they [aprons and gloves] were present there [the patients’ house] so then I used those during the second visit.” (respondent number 16, general practitioner).*

In extramural care, for instance in home-based care, a specialized infection prevention employee was often lacking. So, home-based care nurses had to find out the necessary infection prevention measures themselves. Multiple care providers indicated that they would trust information shared by a specialized employee from the hospital the most. However, the hospital is not always involved in diagnostics and care for these patients. In some interviews, it appeared that care providers were completely dependent on the information that was shared with them by specialized employees, whereas the sender of that information was not always aware of his/her position and relevance in sharing information.

*“By now I know some [resistant bacteria] by heart, but I don’t know this one [CPE] top of mind. So I completely rely on the advise of the medical microbiologist and the infection prevention mostly.” (respondent number 13, physician hospital).*

##### Motivation for applying infection prevention measures

The interviews showed that the level of motivation of care providers to adhere to infection prevention measures differed. Factors that increased care providers motivation to apply infection prevention measures were taking responsibility for their own health, for other patients or for (pregnant) colleagues. However, factors that undermined care providers motivation to apply infection prevention measures were also reported. Some care providers did not see the relevance of the measures and multiple care providers indicated that the infection prevention measures negatively impacted the treatment of patients since they were restricted in moving around through the institution or in exercising (for instance in revalidation care). This subsequently lead to irritation and lack of understanding of measures by patients.

*“It [infection prevention measures] often results in anger, irritation. We see revalidation programs that turn out different than normally. Patients can’t always go to the physical therapist when they want to, or exercise themselves or do this or do that.” (respondent number 7, physician revalidation center).*

Furthermore, multiple respondents indicated that differences in infection prevention measures between settings (admitted to hospital vs. outpatient hospital visit), between care providers and between care providers and family members caused confusion and lead to incomprehension for the advised measures.

*“We [care providers of the patient] were surprised that, according to the infection prevention specialists, this [outpatient hospital visit] was allowed without isolation precautions.” (respondent number 12, physician hospital).*

Critique among healthcare providers also arose when different measures were advised for the period a MDR bacterium was only suspected (for instance when a patient had recently been hospitalized abroad) and following the CPE/CRA-positive diagnosis. This could for example result in the shift from contact isolation to strict isolation when CPE/CRA-positivity was confirmed. Care providers did not always seem to realize that part of the infection prevention measures were instituted to protect other patients. In a few other cases, care providers expressed concern because they believed that the intensity of infection prevention measures was too low and they were skeptical about the effectiveness of the measures.

*“I believe that, despite everything we know about why she [the patient] is in isolation, we should wear protective clothing at all times … because unconsciously you carry it [CPE] with you … I think that both nurses and visitors should adhere to the same rules, because unconsciously it [CPE] is present between the sheets and it [CPE] appears everywhere.” (respondent number 1, nurse revalidation center).*

#### Knowledge and beliefs

The interviews revealed a contradiction in the perceived severity of CPE/CRA-positivity and divergent beliefs on CPE/CRA. Many care providers seemed to take CPE/CRA-positivity seriously and were motivated to enhance their knowledge about CPE/CRA prevention. However, their confidence in measures was limited: multiple respondents indicated that pregnant or vulnerable colleagues, and colleagues with small children were deliberately not involved in the care for CPE/CRA-positive patients.

*“But also fear for if I also have this bacterium and I am unaware of that, how will that go at home? What do I get from that? You know, I rather not have that, because I have a small child at home and I have family members that are pregnant, you know?” (respondent number 17, nurse revalidation center).*

*“Colleagues that feel sick or are vulnerable in terms of health, or we have a colleague that is pregnant, they are definitely not employed [in the care for CPE-positive patients].” (respondent number 2, nurse home- based care).*

Additionally, multiple respondents indicated the need for CPE/CRA-specific guidelines, including specific advice for the aforementioned pregnant or vulnerable colleagues, and colleagues with small children.

*“There are no specific guidelines for us [general practitioners], so I think that is a real issue. Mainly for practical reasons: how do you deal with that [CPE positive patients], how long does it [CPE positivity] take, is there a limit for the contact isolation or should it be continued completely?” (respondent number 16, general practitioner).*

Often, there were initiatives of CPE-related training or education when CPE/CRA-positive patients were admitted to the institution. Some care providers indicated that the extra attention for and information about CPE/CRA by means of education was appreciated, took away concern, and that the training matched the expectations and needs.

*“We had clinical training by the hygienists about that [CPE] … it was very clear why certain measures had to be taken … A clinical training takes away stress, concern and questions … They explained it to us in understandable language, for all levels of personnel that are employed at our institution. So for individuals working in the kitchen, but also for all nurses.” (respondent number 17, nurse revalidation center).*

In some cases, the follow-up policy for a CPE/CRA-positive patient was clear and in these cases care providers shared extensive information about the patient, the procedures and infection prevention advices with subsequential care providers.

*“Yes, I wrote a letter about what happened during the admission and what the result of the cultures was and how we deal with that and what the advices are to implement there [at subsequent institution].” (respondent number 9, medical fellow hospital).*

In one case, infection prevention materials were given to the patient for the home-based care employees to enable infection prevention measures.

“*We always provide the patient with a few aprons for the home-based care employees.” (respondent number 14, healthcare assistant revalidation center).*

Several other inadequacies in the CPE/CRA knowledge of care providers were also observed. Some care providers thought that CPE/CRA was a virus, others thought that CPE/CRA is abundantly present on and around a CPE/CRA-positive patient (for instance on skin and other body parts, in clothes, between sheets or in the entire room) and that transmission can occur in numerous ways (for instance when hugging family, shaking hands or standing in an elevator).

“*When such a man [CPE positive patient] comes outside, and he carries such a dangerous organism and he is standing in an elevator … and he goes home and hugs his wife, to what extent is that dangerous? And I don’t know much about that in specifically this bacteria [CPE].” (respondent number 12, physician hospital).*

Many care providers were unaware of the follow-up policy for CPE/CRA-positive patients in terms of both follow-up testing and sharing of information with simultaneously involved or future care providers when a patient was discharged. Often the CPE/CRA status of the patient was communicated when transferring the patient to a subsequent care provider, but advice on how to act was not.

*“I do think there is a clear policy in the hospital. But what happens when a patient is discharged … Yes, then you are curious: what about all that?” (respondent number 12, physician hospital).*

*“Well, he [the patient] and his wife were able to tell what was wrong with him very well themselves. Yes, they talked a lot about the bacterium that he carried, so I am not sure whether my colleagues called the hospital where he was also a patient upon discharge, but if we did not tell them, I can guarantee you that they [the patient and his wife] would announce it themselves.” (respondent number 17, nurse revalidation center).*

Almost all respondents indicated that guidelines or protocols for MDR bacteria exist within the organization. However, specific guidelines and protocols for CPE/CRA-positivity are often lacking. Even when guidelines and protocols are present within an organization, care providers are frequently not certain where these guidelines can be found. In institutional care, care providers often indicated to contact infection prevention specialists when their knowledge concerning CPE/CRA-positivity and infection prevention measures was insufficient. Almost all respondents indicated the need for extra information and education concerning (the care for) CPE/CRA-positive patients. Some care providers preferred information solely about the bacteria and mechanisms of resistance, and others preferred information merely about the required infection prevention precautions (why do measures need to be taken vs. what measures need to be taken).

## Discussion

The purpose of this study was to firstly gain insight into the frequency of CPB-positive patients in the Dutch provinces of NH and FL by means of a quantitative component. Secondly, by means of a qualitative component, we aimed to obtain a deeper understanding of the communication process between healthcare providers during transfers of CPB-positive patients and explore possible communication-related risk situations for the spread of CPB. For the quantitative section of this study. a total of 64 patients were reported in NH and FL from February 2018 to February 2019. Our study shows that in case of the 18 healthcare providers included in the qualitative section of the study, the information regarding a CPE/CRA-positive patient being transferred was mostly shared timely. However, methods for the transference of information were diverse and in almost all cases, the transference of information was not standardized. Many care providers could not exactly recall how and by who the information was shared with them. The motivation to adhere to precautionary measures in case of a CPE/CRA-positive patient differed between care providers. Factors that enhance motivation were taking responsibility for the health of other patients, for (pregnant) colleagues and for ones own health. Factors that reduce motivation were not acknowledging the relevance of the precautionary measures, a perceived negative impact of the precautionary measures on the patients’ recovery, differences in precautionary measures between healthcare settings and incomprehension for the possible shift in previous advised precautionary measures. Most care providers had not encountered CPE/CRA-positive patients before and in the majority of cases, the follow-up policy for the CPE/CRA-positive patient was unclear. Almost all care providers indicated that they took CPE/CRA-positivity seriously and specified that they felt the need to obtain more information concerning CPE/CRA-positivity.

Since CPE are still relatively rare in the Netherlands and other European countries [3, 17, 19, 20, 22], based on estimates of medical microbiologists we expected to find 15 to 20 CPE-positive patients in NH and FL in one year. Even when not taking the 22 related outbreak patients into account, the total of 28 other CPE-positive patients that were reported was substantially higher than expected. As the prevalence of CPE is rising worldwide [3, 33–35], our findings might suggest that the prevalence is also rising in the Netherlands. Indeed, national surveillance data from ISIS-AR show that confirmed non-susceptibility in *E. coli* and *K. pneumoniae* isolates was low but slightly increasing over the past 5 years (0.05 and 0.52% in 2018, respectively) [22].

In line with our expectations, we found that experience with CPE/CRA-positivity and applicability of precautionary measures was more present in the ‘cure’ settings, since AMR has been a subject of interest in these settings for a longer time and consequences of AMR for the vulnerable (inpatient) population can be severe. However, in public health, or ‘care’ settings, we found that healthcare providers have not been confronted with MDR bacteria regularly, so experience with CPE/CRA and applicability of precautionary measures was found to be lower. Our study showed that for most care providers, this was the first time they encountered a CPE/CRA-positive patient and that the transference of information regarding a CPE/CRA-positive patient was not clearly arranged.

Also, adequate knowledge on CPE/CRA was often lacking. Multiple care providers indicated the need for more information on CPE/CRA. Often, a single moment of extra education for healthcare providers was organized within an institution when a CPE/CRA-positive patient was encountered. Even though the training met expectations and needs of care providers, the single moment of education could lead to a delay in application of adequate measures by the care providers from the moment a CPE/CRA-positive patient was diagnosed. These findings advocate the need for structurally training medical staff on CPE/CRA. To maximize understanding and optimize compliance with infection prevention measures, the training should emphasize the substantiated and consciously formulated differences between guidelines for different healthcare settings. Training on CPE/CRA could actively involve care providers, for instance through e-learnings on CPE/CRA. E-learnings are accessible at all times and can reduce delay in education when a CPE/CRA-positive patient is admitted or encountered. Also, to guarantee sufficient knowledge is present within an institution at all times, we suggest to appoint one or several designated individuals within healthcare institutions that possess knowledge on CPE/CRA and who actively inform others within the institution in case of CPE/CRA-positive patients. Our findings on the importance of adequate knowledge and perceived severity among healthcare providers correlate with findings from a study exploring barriers and enablers to MRSA admission screening in hospitals [36] and a study into the acceptability of screening for CPE [37].

Furthermore, it is advised to standardize patient transfer information (for instance through electronic transfer dossier) in which it is obliged to indicate whether or not a patient carries a resistant bacterium. Finally, these findings indicate the need for uniform, transmural agreements concerning CPE/CRA-positive patients. Constituting transmural agreements has been described as one of the tasks for the 10 regional antimicrobial resistance networks in the Netherlands. These networks were commissioned by the Dutch Ministry of Health, Welfare and Sport in 2016 and have been formally implemented in May 2017.

Various care providers specified the need for the development of a CPE/CRA-specific guideline by the Dutch General Practitioner Society (in Dutch: Nederlandse Huisartsen Genootschap (NHG)) to help general practitioners in optimizing care for CPE/CRA-positive patients. However, since NHG guidelines are specifically for general practitioners, we additionally advocate for the expansion of the multidisciplinary guideline on highly resistant microorganisms by the National Coordination of Infectious Disease Control (in Dutch: Landelijke Coördinatie Infectieziektebestrijding (LCI)) which is applicable for all care providers. This guideline should include information and hands-on advise for care providers on the diagnosis, treatment and care for CPE/CRA-positive patients and their families. The guideline should emphasize why differences in infection prevention measures exist between settings and bacteria since incomprehension might lead to non compliance.

Moreover, we believe that an integrated national website on CPE/CRA should be developed. This website should include clear patient information folders and a realistic risk assessment about the severity of CPE/CRA-positivity and infections for both patients, household contacts/visitors, and care providers. The information on the website should be comprehensible for patients of all educational levels. This recommendation correlates with findings from a recent study into patient experiences concerning hospital screening for CPE, which highlighted the need for access to clear patient information on CPE [38].

We believe that the major strength of our study is the mixed-methods design. This has allowed us to both provide insight into the number of CPB-positive patients within the provinces of NH and FL and explore themes within communication of healthcare providers that could potentially influence the transmission of CPB within and between healthcare institutions. However, our study also had several limitations. Firstly, the diagnosing laboratories of NH and FL voluntarily reported CPE-positive patients. Only one participating laboratory automated the reporting of CPE-positive patients, all other laboratories manually reported CPE-positive patients to the research team. This might have resulted in not all diagnosed patients being reported to the research team and an underestimation of the true number of CPE-positive patients in NH and FL. However, the number of patients reported to the research team did not substantially deviate from the number of patients reported from the same laboratories to the National Institute for the Public Health and the Environment (RIVM) for surveillance for those regions. Nonetheless, reporting of CPE-positive patients to the RIVM is also on a voluntary basis and not all laboratories that provide diagnostics for the NH and FL region participate in this surveillance. It is therefore likely that underreporting and thereby an underestimation of the true number of CPE-positive patients in NH and FL has actually occurred. An underestimation of the true number of CRA and CRP patients has most likely occurred, since only two laboratories reported these bacteria and they are included in the study as an additional finding. Also, we were unable to include 20 out of 29 patients that were eligible for inclusion in the qualitative section. The 20 patients that were eligible but were not included were more often female and were younger compared to the eligible patients that were included, which could have lead to participation bias. However, we do not believe that the care for the eligible, not included patients differs from the care for the eligible, included patients and therefore the effect of the participation bias on the study results is expected to be negligible. Furthermore, partly due to the limited inclusion period, we were only able to include healthcare providers of 9 patients in the qualitative section of the study and we were unable to include multiple healthcare providers for 4 patients. However, we do believe that the 20 interviews concerning the 9 included patients provided us with valuable insights into communication of care providers concerning CPE/CRA-positive patients. It is expected that healthcare providers will be more inclined to participate in comparable studies since CPE-positivity became mandatory notifiable on July 1st 2019. Also, the time interval for reporting CPE-positive patients will decrease from July 1st 2019 because the accepted period of reporting has been legally maximized at one working day. Moreover, mainly due to the mixed-methods design of the study and the limited inclusion period, a relatively limited content analysis of the qualitative data was performed resulting in limited conceptual interpretation. However, we do believe that valuable information was derived from the interviews with the healthcare providers. Future strongly designed qualitative studies could be performed to confirm and validate our findings. Due to the semi-structured character of the interviews, we cannot exclude interperson variability in the interviews. However, we tried to minimize variability by simultaneously providing both research nurses with interview instructions, during which questions were addressed and uncertainties were resolved. Furthermore, both nurses made use of the same interview guide.

## Conclusions

CPE-positivity occurred more frequently than expected in the Dutch provinces of NH and FL. Most care providers are not used to caring for CPE/CRA-positive patients and adequate knowledge concerning CPE/CRA is often lacking. Standardizing the transference of information concerning CPE/CRA-positive patients could improve communication between healthcare providers and thereby decrease the risk of CPE/CRA transmission during patient transfers. This would ideally include at least automated timely data exchange (among others of the CPE/CRA status and easily accessible information on necessary precautionary measures) between institutions before transfer actually takes place. Furthermore, formal and mutually consented transmural agreements could contribute to optimizing communication about CPE/CRA-positive patients. Additionally, care providers’ knowledge about CPE/CRA and advised precautionary measures could be enhanced by means of E-learnings, and a national website on CPE/CRA could offer information to both care providers and CPE/CRA-positive patients. Finally, one or several designated employees for CPB or MDR bacteria in general within an institution should be responsible for maintaining and sharing knowledge (also during MDR cases or outbreaks) within the institution. In our opinion, the findings of our study are transferable to other regions of the Netherlands. Transferability to other countries is dependent on the healthcare infrastructure (for instance whether cure and care domains are separated as is the case in the Netherlands). Future qualitative studies could deepen our understanding of the importance of communication between healthcare providers in battling AMR worldwide. Furthermore, future studies should focus on the feasibility of the implementation of proposed recommendations (drivers and barriers) in various healthcare settings and institutions.

## Supplementary information

**Additional file 1.** Interview guide Interview guide belonging to the manuscript “Perceived barriers and enablers for preventing the spread of carbapenem producing gram-negative bacteria during patient transfers: a mixed methods study among health care providers”.

## Data Availability

The data used and/or analyzed during the current study are available from the corresponding author on reasonable request.

## Abbreviations

AMR: Antimicrobial resistance
CIM: Carbapenem inactivation method
CPB: Carbapenem-producing gram-negative bacteria
CPE: Carbapenem-producing *Enterobacteriaceae*
CRA: Carbapenem-resistant *Acinetobacter spp.*
CRP: Carbapenem-resistant *Pseudomonas aeruginosa*
EPD: Electronic patient dossier
FL: Flevoland
IQR: Inter quartile range
ISIS-AR: Infectious diseases Surveillance System-Antibiotic Resistance
LCI: National Coordination of Infectious Disease Control
MDR: Multidrug-resistant
MIC: Mean inhibitory concentration
MRSA: Methicillin-resistant *Staphylococcus aureus*
NH: Noord-Holland
NHG: Dutch General Practitioner Society
NVMM: Dutch Society for Medical Microbiology
PHS: Public Health Service
RIVM: National Institute for Public Health and the Environment
SO-ZI/AMR: National early warning and response meeting of hospital-acquired infections and antimicrobial resistance
WHO: World Health Organization

## References

[1] O'Neill J. Tackling drug-resistant infections globally: final report and recommendations. The review on antimicrobial resistance 2016.

[2] Factsheet on Antibiotic Resistance World Health Organization; 2018 [Available from: http://www.who.int/news-room/fact-sheets/detail/antimicrobial-resistance. Accessed 31 July 2018.

[3] Cassini A, Hogberg LD, Plachouras D, Quattrocchi A, Hoxha A, Simonsen GS (2019). Attributable deaths and disability-adjusted life-years caused by infections with antibiotic-resistant bacteria in the EU and the European economic area in 2015: a population-level modelling analysis. Lancet Infect Dis.

[4] Surveillance of antimicrobial resistance in Europe 2017. European Centers for Disease Prevention and Control (ECDC); 2017.

[5] Brauer R, Ruigomez A, Downey G, Bate A, Garcia Rodriguez LA, Huerta C (2016). Prevalence of antibiotic use: a comparison across various European health care data sources. Pharmacoepidemiol Drug Saf.

[6] Schouten C, Bruins B. State of affairs concerning antibiotic policy in livestock. Hague: Ministry of Agriculture, Nature and Food Quality; 2017.

[7] Wertheim HF, Vos MC, Boelens HA, Voss A, Vandenbroucke-Grauls CM, Meester MH (2004). Low prevalence of methicillin-resistant Staphylococcus aureus (MRSA) at hospital admission in the Netherlands: the value of search and destroy and restrictive antibiotic use. J Hosp Infect.

[8] Overview of existing guidelines MRSA and HRMO by healthcare institution/healthcare provider (in Dutch: Overzicht beschikbare richtlijnen MRSA en BRMO per zorgverlenende instelling/zorgverlener): National Institute for Public Health and the Environment (RIVM); 2015 [Available from: https://lci.rivm.nl/sites/default/files/2017-10/1.%20Overzicht%20beschikbare%20richtlijnen%20BRMO%20en%20MRSA.pdf. Accessed 13 Aug 2018.

[9] Arcilla MS, van Hattem JM, Haverkate MR, Bootsma MCJ, van Genderen PJJ, Goorhuis A (2017). Import and spread of extended-spectrum beta-lactamase-producing Enterobacteriaceae by international travellers (COMBAT study): a prospective, multicentre cohort study. Lancet Infect Dis.

[10] Dautzenberg MJ, Ossewaarde JM, de Kraker ME, van der Zee A, van Burgh S, de Greeff SC, et al. Successful control of a hospital-wide outbreak of OXA-48 producing Enterobacteriaceae in the Netherlands, 2009 to 2011. Euro Surveill. 2014;19(9). 10.2807/1560-7917.es2014.19.9.20723 24626209

[11] Weterings V, Zhou K, Rossen JW, van Stenis D, Thewessen E, Kluytmans J (2015). An outbreak of colistin-resistant Klebsiella pneumoniae carbapenemase-producing Klebsiella pneumoniae in the Netherlands (July to December 2013), with inter-institutional spread. Eur J Clin Microbiol Infect Dis.

[12] Bastiaens GJH, Cremers AJH, Coolen JPM, Nillesen MT, Boeree MJ, Hopman J (2018). Nosocomial outbreak of multi-resistant Streptococcus pneumoniae serotype 15A in a Centre for chronic pulmonary diseases. Antimicrob Resist Infect Control.

[13] NH-FL AZA. Regional risk profiles antimicrobial resistance [Available from: https://www.abrzorgnetwerknhfl.nl/activiteiten/regionaal-risicoprofiel/. Accessed 20 Oct 2018.

[14] WHO priority pathogens list for R&D of new antibiotics: World Health Organization; 2017 [Available from: http://www.who.int/news-room/detail/27-02-2017-who-publishes-list-of-bacteria-for-which-new-antibiotics-are-urgently-needed.

[15] The Australian Commission on Safety and Quality in Health Care. Recommendations for the control of carbapenemase-producing Enterobacteriaceae (CPE): A guide for acute health care facilities. Infection, Disease & Health. 2017;22;159-86.

[16] van Duin D, Doi Y (2017). The global epidemiology of carbapenemase-producing Enterobacteriaceae. Virulence..

[17] Canton R, Akova M, Carmeli Y, Giske CG, Glupczynski Y, Gniadkowski M (2012). Rapid evolution and spread of carbapenemases among Enterobacteriaceae in Europe. Clin Microbiol Infect.

[18] Grundmann H, Glasner C, Albiger B, Aanensen DM, Tomlinson CT, Andrasevic AT (2017). Occurrence of carbapenemase-producing Klebsiella pneumoniae and Escherichia coli in the European survey of carbapenemase-producing Enterobacteriaceae (EuSCAPE): a prospective, multinational study. Lancet Infect Dis.

[19] Albiger B, Glasner C, Struelens MJ, Grundmann H, Monnet DL. European Survey of Carbapenemase-Producing Enterobacteriaceae working g. Carbapenemase-producing Enterobacteriaceae in Europe: assessment by national experts from 38 countries, May 2015. Euro Surveill. 2015;20(45). 10.2807/1560-7917.ES.2015.20.45.30062 26675038

[20] Trepanier P, Mallard K, Meunier D, Pike R, Brown D, Ashby JP (2017). Carbapenemase-producing Enterobacteriaceae in the UK: a national study (EuSCAPE-UK) on prevalence, incidence, laboratory detection methods and infection control measures. J Antimicrob Chemother.

[21] Leenstra T, Bosch T, Vlek AL, Bonten MJM, van der Lubben IM, de Greeff SC (2017). Carbapenemase producing Enterobacteriaceae in the Netherlands: unnoticed spread to several regions. Ned Tijdschr Geneeskd.

[22] Consumption of antimicrobial agents and antimicrobial resistance among medically important bacteria in the Netherlands (NethMap). Dutch National Institute for Public Health and the Environment; 2019.

[23] Nordmann P (2014). Carbapenemase-producing Enterobacteriaceae: overview of a major public health challenge. Med Mal Infect.

[24] Sheu CC, Chang YT, Lin SY, Chen YH, Hsueh PR (2019). Infections caused by Carbapenem-resistant Enterobacteriaceae: an update on therapeutic options. Front Microbiol.

[25] Villegas MV, Pallares CJ, Escandon-Vargas K, Hernandez-Gomez C, Correa A, Alvarez C (2016). Characterization and clinical impact of bloodstream infection caused by Carbapenemase-producing Enterobacteriaceae in seven Latin American countries. PLoS One.

[26] Bruins B. Kamerbrief voortgang antibioticaresistentie (in Dutch). In: Ministry of Health WaS, editor. The Hague2018.

[27] Highly Resistant Micro Organisms, particularly Carbepenem Producing Enterobacteriaceae (CPE) guideline (in Dutch: Bijzonder resistente microorganismen (BRMO), in het bijzonder carbapenemproducerende Enterobacteriaceae (CPE) Richtlijn. Dutch Ministry of Health, Welfare and Sport; 2019.

[28] van Loon K, Voor In, t Holt AF, Vos MCA. Systematic Review and Meta-analyses of the Clinical Epidemiology of Carbapenem-Resistant Enterobacteriaceae. Antimicrob Agents Chemother. 2018;62(1). 10.1128/AAC.01730-17 PMC5740327 29038269

[29] NVMM Guideline. Laboratory detection of highly resistant microorganisms (HRMO). Dutch Society for Medical Microbiology (in Dutch: Nederlandse Vereniniging voor de Medische Microbiologie (NVMM)); 2012.

[30] Guzel O, Guner EI (2009). ISO 15189 accreditation: requirements for quality and competence of medical laboratories, experience of a laboratory I. Clin Biochem.

[31] van der Zwaluw K, de Haan A, Pluister GN, Bootsma HJ, de Neeling AJ, Schouls LM (2015). The carbapenem inactivation method (CIM), a simple and low-cost alternative for the Carba NP test to assess phenotypic carbapenemase activity in gram-negative rods. PLoS One.

[32] Smith JA, Smith JA, Harre R, Langenhove LV (1995). Semi-structured interviewing and qualitative analysis. Rethinking methods in psychology.

[33] Logan LK, Weinstein RA (2017). The Epidemiology of Carbapenem-Resistant Enterobacteriaceae: The Impact and Evolution of a Global Menace. J Infect Dis.

[34] Nordmann P, Poirel L (2014). The difficult-to-control spread of carbapenemase producers among Enterobacteriaceae worldwide. Clin Microbiol Infect.

[35] Nordmann P, Naas T, Poirel L (2011). Global spread of Carbapenemase-producing Enterobacteriaceae. Emerg Infect Dis.

[36] Currie K, King C, McAloney-Kocaman K, Roberts NJ, MacDonald J, Dickson A (2019). Barriers and enablers to meticillin-resistant Staphylococcus aureus admission screening in hospitals: a mixed-methods study. J Hosp Infect..

[37] Currie K, King C, McAloney-Kocaman K, Roberts NJ, MacDonald J, Dickson A (2018). The acceptability of screening for Carbapenemase producing Enterobacteriaceae (CPE): cross-sectional survey of nursing staff and the general publics' perceptions. Antimicrob Resist Infect Control.

[38] King C, Grandison T, Cawthorne J, Currie K. Patient experience of hospital screening for carbapenemase-producing Enterobacteriaceae: a qualitative study. J Clin Nurs. 2019. 10.1111/jocn.14982 31240778

