# Correction to: Perceived barriers and enablers for preventing the spread of carbapenemase-producing gram-negative bacteria during patient transfers: a mixed methods study among healthcare providers

**DOI:** 10.1186/s12879-020-4788-3

**Published:** 2020-01-22

**Authors:** Eline van Dulm, Wendy van der Veldt, Katja Jansen-van der Meiden, Gerry van Renselaar, Lian Bovée, Jeanette Ros, Udi Davidovich, Yvonne van Duijnhoven

**Affiliations:** 1 Department of Infectious Diseases, Public Health Service Amsterdam, Nieuwe Achtergracht 100, 1018 WT Amsterdam, the Netherlands; 2 Department of Infectious Diseases, Public Health Service Kennemerland, Haarlem, the Netherlands; 3 Department of Infectious Diseases, Public Health Service Flevoland, Lelystad, the Netherlands

**Correction to: BMC Infectious Diseases (2019)**

**https://doi.org/10.1186/s12879-019-4684-x**

After publication of the original article [1], we have noticed that the word ‘Carbapenem-producing’ should be replaced with ‘Carbapenemase-producing’.

## References

[1] van Dulm E (2019). Perceived barriers and enablers for preventing the spread of carbapenem producing gram-negative bacteria during patient transfers: a mixed methods study among healthcare providers. BMC Infect Dis.

