# Supplementary material for: Perceived barriers and enablers for preventing the spread of carbapenem producing gram-negative bacteria during patient transfers: a mixed methods study among healthcare providers
Source: BMC Infect Dis. 2019 Dec 11;19:1050. doi: 10.1186/s12879-019-4684-x (PMC6907261; doi:10.1186/s12879-019-4684-x)
Supplement: Supplementary file 1 — Additional file 1. Interview guide Interview guide belonging to the manuscript “Perceived barriers and enablers for preventing the spread of carbapenem producing gram-negative bacteria during patient transfers: a mixed methods study among health care providers”. [file 12879_2019_4684_MOESM1_ESM.docx]

**Research question:**

What are potential risk situations for the spread of CPB in communication between healthcare providers during transfers of CPB-positive patients?

**Interview questions:**

- Introduction:
  - Can you explain me your role in the care for this patient?
  - When were you first involved in the care for this patient?
- Information exchange during patient transfers:
  - Can you indicate how you have been informed about this patient (by whom, in what manner (written/by telephone/digital), when (before/after the transfer/the same day))
  - Was it immediately clear that you were dealing with a CPB-positive patient?
- Precautionary measures:
  - What precautionary measures have been taken for this patient?
  - Can you indicate the grounds on which these decisions have been made?
  - Have you encountered any barriers in applying these measures?
- Availability of guidelines:
  - Are there any precautionary measure guidelines within your institution that you have consulted for this patient?
  - If yes, are these guidelines easy to apply?
- Knowledge of healthcare provider:
  - Were you already familiar with CPB?
  - Would you need additional information and advice on CPB and necessary precautionary measures?
  - If yes, what kind of information/advice?
  - From whom and in what manner would you prefer to receive additional information and advice?
  - When would you prefer to receive additional information and advice?
- Other healthcare providers:
  - Are there any other healthcare providers involved in the care for this patient?
